# Pan-European Chikungunya surveillance: designing risk stratified surveillance zones

**DOI:** 10.1186/1476-072X-8-61

**Published:** 2009-10-31

**Authors:** Natasha Tilston, Chris Skelly, Phil Weinstein

**Affiliations:** 1 ieSim, London, United Kingdom, formerly at the Institute for the Environment, Brunel University West London, Uxbridge, UK; 2 School of Population Health, The University of Queensland, Brisbane, Australia

## Abstract

The first documented transmission of Chikungunya within Europe took place in Italy during the summer of 2007. Chikungunya, a viral infection affecting millions of people across Africa and Asia, can be debilitating and no prophylactic treatment exists. Although imported cases are reported frequently across Europe, 2007 was the first confirmed European outbreak and available evidence suggests that *Aedes albopictus *was the vector responsible and the index case was a visitor from India. This paper proposed pan-European surveillance zones for Chikungunya, based on the climatic conditions necessary for vector activity and viral transmission. Pan-European surveillance provides the best hope for an early-warning of outbreaks, because national boundaries do not play a role in defining the risk of this new vector borne disease threat. A review of climates, where Chikungunya has been active, was used to inform the delineation of three pan-European surveillance zones. These vary in size each month across the June-September period of greatest risk. The zones stretch across southern Europe from Portugal to Turkey. Although the focus of this study was to define the geography of potential surveillance zones based on the climatic limits on the vector and virus, a preliminary examination of inward bound airline passengers was also undertaken. This indicated that France and Italy are likely to be at greater risk due to the number of visitors they receive from Chikungunya active regions, principally viraemic visitors from India. Therefore this study represents a first attempt at creating risk stratified surveillance zones, which we believe could be usefully refined with the use of higher resolution climate data and more complete air travel data.

## Introduction

The arbovirus Chikungunya is transmitted to humans primarily by the mosquito species *Aedes aegypti *and *Aedes albopictus *[1-3]. Although the symptomology of this infection was first noticed in the late 1770s, the pathogen became recognised only in 1952 when the virus was isolated during a Dengue outbreak in Tanzania, East Africa [1,3-5]. Chikungunya shares clinical similarities with Dengue and reports have shown both viruses occurring simultaneously in patients [4,6-11]. Therefore it is possible that Dengue could have (and may still) mask cases of Chikungunya. The name, Chikungunya, originates from the Makonde dialect of Tanzania, and refers to the patient's contorted posture as a result of severe joint pains. Records also show that the same disease has been referred to in India as Aakdaya and Maakdya, meaning "stiff-man" and "monkey-like" respectively [3,12,13].

Numerous deaths from Chikungunya were recorded between 2001 and 2007 in Asia and further gastroenteritis, neurological complications and foetal deaths were also recorded [14-17]. Although these conditions are rare and Chikungunya is not usually life threatening, it does cause extreme discomfort and mobility problems which can last up to several months [18]. The Italian outbreak in 2007, Europe's first outbreak, confirmed the possibility of such infectious diseases becoming global due to air travel [19,20]. Dengue, also spread by the same vectors as Chikungunya, is therefore a coincident risk, with the potential to cause even more morbidity. According to the European Centre for Disease Prevention and Control (ECDC), the risk is serious because with an already established vector population, larger outbreaks would increase the difficulty in eradicating the disease in Europe [20].

**Figure 1:**
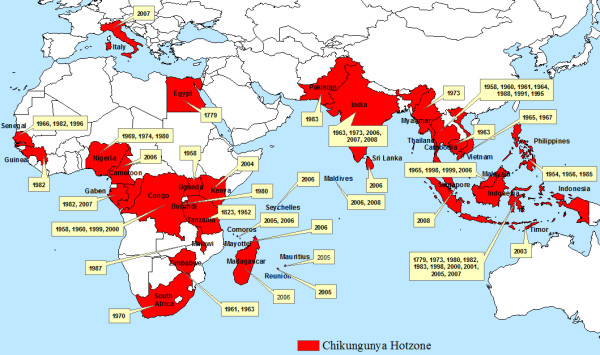
**Chikungunya active transmission regions established from published data illustrate our geographic and temporal knowledge of previous outbreaks**.

Endemic and epidemic Chikungunya is geographically spread over Africa, South East Asia, the Indian Subcontinent and the Western Pacific (Figure 1) [2,11,21]. The first known local transmission of Chikungunya in Europe occurred in the Emilia-Romagna Province of Italy in 2007 [1,22]. This outbreak raised concern because the vector, *Aedes albopictus*, is already established over much of southern Europe [22-25]. In addition, a large number of air travellers from Chikungunya infected regions enter Europe each year [2,10,26]. Numerous studies have already begun to describe the problem, especially in regards to the ecology of *Aedes albopictus *[26-31], which the WHO describes as capable of surviving cold climates and thereby extending the current range of Chikungunya [21]. In this paper, we define the areas across Europe where Chikungunya transmission may occur based on the climatic limitations of the vector and viral transmission. On this basis we create stratified surveillance zones, varying over space and time, that could be used as the basis for a more efficient and effective surveillance programme. We supplement these findings with a preliminary examination of airline passenger data to show the most probable routes of viral introductions into Europe. We integrate our findings into a set of stratified Pan-European surveillance zones.

## Methods

### An Epidemiological model of Chikungunya in Europe

A number of elements must be present for Chikungunya outbreaks to occur in Europe:

***i. A competent vector***: Chikungunya in Europe is most likely to be transmitted by *Aedes albopictus*, since it has already proven its competency during the Italian outbreak in 2007. Other competent vectors may also exist, for example *Aedes vittatus *and *Aedes caspius *that are established in France, have transmitted the virus under laboratory conditions [26].

***ii. A susceptible population***: since the European population has not previously been widely exposed to Chikungunya, it can be assumed that the population lacks herd immunity.

***iii. Environmental conditions suitable for transmission of the virus***: the present study focuses only on the temperatures required for Chikungunya transmission.

***iv. Initial exposure of the susceptible population to the virus***: to initiate an outbreak of Chikungunya within Europe, the susceptible population must be exposed to the virus, and this exposure will most likely come from viraemic passengers travelling into Europe from affected areas (and possibly also through the importation of infected mosquitoes, which we do not consider in this paper). We undertake a preliminary analysis of air passenger data for people inbound from known Chikungunya hotspots around the world.

### The climatic scope of Chikungunya transmission

**Figure 2:**
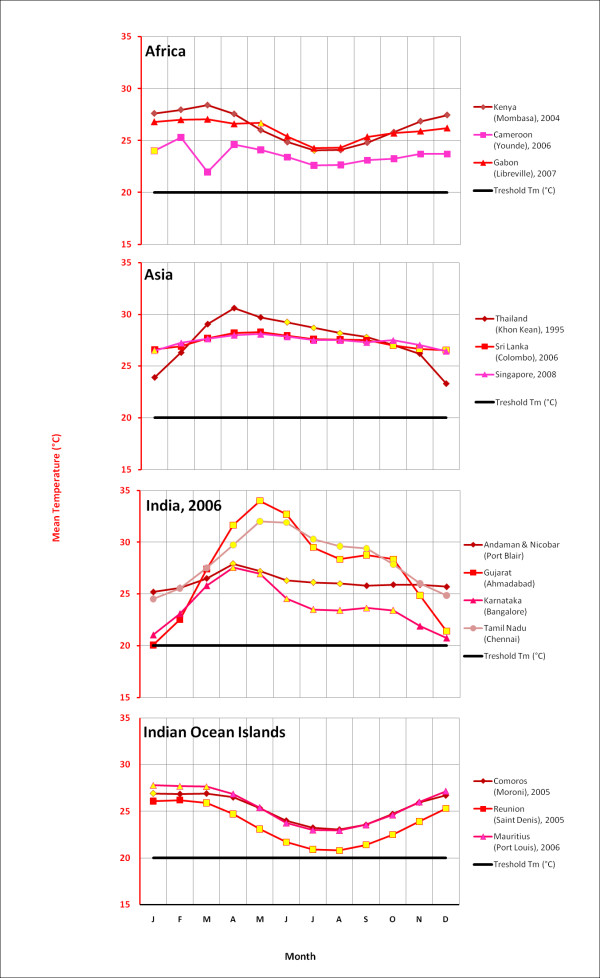
**Temperature statistics for a selection of Chikungunya active regions**. The yellow markers indicate months during which outbreaks occur.

We examined the monthly average temperature of cities that experience Chikungunya outbreaks and identified the temperature range during the months when outbreaks occurred (Figure 2). This analysis indicates that average monthly temperatures need to be 20°C or higher before outbreaks occur. Fourteen cities in Africa, Asia and the Indian Ocean Islands were selected based on the availability of climate data for the month of an outbreak (Figure 2). Monthly mean temperature data were obtained from the World Meteorological Organization [32-40], World Climate [41], World Travel Guide [42] and Euro weather [43].

**Figure 3:**
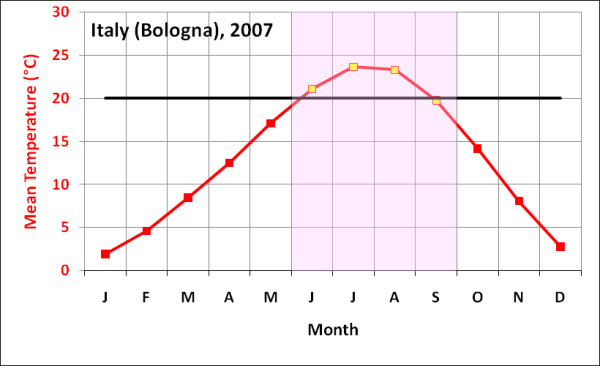
**Temperature and humidity statistics for Italy, the only known site for European transmission of Chikungunya**. Note the yellow markers indicate months during which the outbreak occurred.

The outbreak in Italy started in June when mean monthly temperature is around 22°C and subsided in September as temperatures fell below 20°C (Figure 3), which appears to reconfirm that the chosen temperature range derived from overseas outbreaks is a useful guide. Therefore, we use this as the basis for assuming that Chikungunya transmission occurs mostly in climates with mean monthly temperatures over 20°C. Although there are many limitations to such an approach, the aim is to begin developing guidelines, which can be refined, to indicate probably temperature ranges for Chikungunya transmission.

### Creating surveillance zones

European climate data were obtained from WORLDCLIM version 1.4 [44] at a resolution of 30 arc seconds. This dataset contains monthly average minimum and maximum temperature and precipitation for the time period 1950 - 2000, and is estimated on a cell by cell basis for all of Europe. Based on these data, we identify areas that satisfy the vector's survival criteria: (a) overwintering conditions that must be at or above the 0°C January Isotherm with at least 500 mm annual rainfall [25,45], and (b) monthly mean temperatures of 10°C or more that allow adult *Aedes albopictus *populations to develop. This temperature condition was chosen because observations show that few European mosquito populations are found to be active at temperatures of 8.5°C or less [24,26,46]. However, active populations of *Aedes albopictus *have been found at temperatures as low as 12.6°C [47] and are thought to be able to survive temperatures of at least 10°C [48]. Temperature is also a factor in effective viral transmission by mosquitoes, and we estimate that a mean monthly temperature of 20°C or more is required for Chikugunya transmission, based on observations at known outbreak locations (Figure 2). Using these climatic constraints for vector survivability and viral transmission, we propose three variable pan-European surveillance zones:

***Low priority surveillance zone***: areas of Europe where the main vector, *Aedes albopictus*, has the potential to overwinter and there is at least 500 mm of precipitation, annually. This provides a baseline surveillance zone, because *Aedes albopictus *outside this zone will not be able to maintain a population from one year to the next.

***Medium priority surveillance zone***: areas, that in addition to having the potential for overwintering of mosquito populations, also have seasonal temperatures that support the development of adult *Aedes albopictus *populations.

***High Priority Surveillance Zone***: areas that have the potential to overwinter populations, support the development of adult *Aedes albopictus *populations seasonally and also have sufficiently warm seasonal temperatures to support viral transmission.

Areas outside of the proposed surveillance zones have none of the vector or transmission potential required for local outbreaks to be a serious risk.

### Analysis of air passenger traffic data

Assessment of potential Chikungunya virus importation into Europe from infected regions is estimated from air traffic passenger data. International airports in Chikungunya active regions and their European destinations were identified online [49] and passenger air traffic data obtained from the FlightStats database [50], which provides flight numbers over a 3 day window for all direct flights across the world. Data were accumulated for a one week period (01/09/08 - 07/09/08) for each major airport in regions identified in the literature as having significant outbreaks. The number of passengers is estimated using aircraft capacity. Based on these data, the number of flights and passengers travelling inbound to Europe from areas of Chikungunya activity each a year are estimated. Also, an approximate number of passengers that might be viraemic were very crudely estimated using the proportion of the population thought to be affected in each country of concern (or sub-region in the case of India) [8,11,22,51-61].

## Results

**Figure 4:**
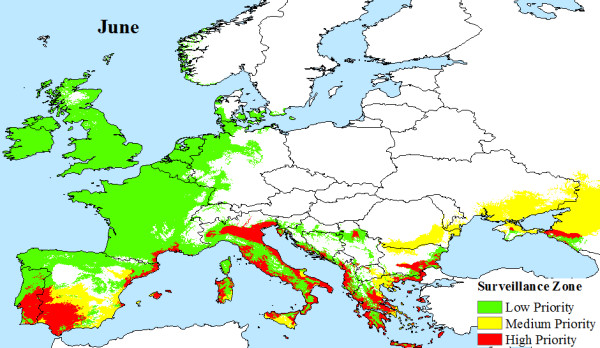
**Proposed European Chikungunya Surveillance Zones for June**.

**Figure 5:**
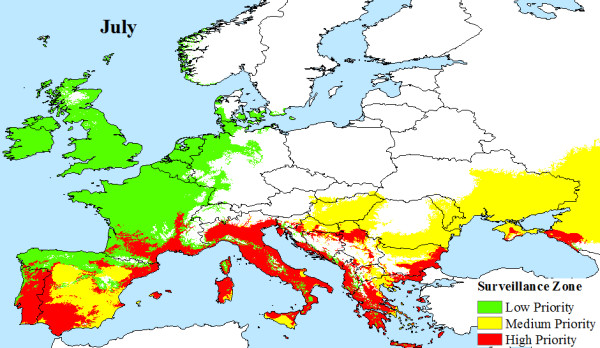
**Proposed European Chikungunya Surveillance Zones for July**.

**Figure 6:**
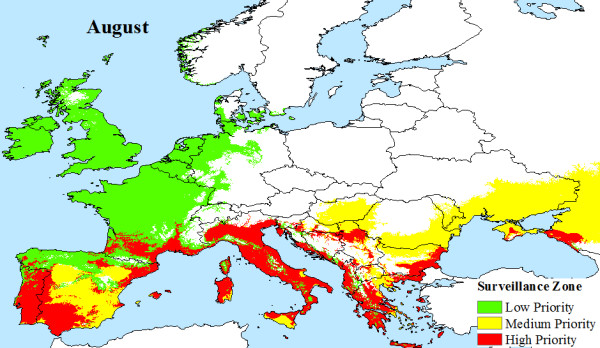
**Proposed European Chikungunya Surveillance Zones for August**.

**Figure 7:**
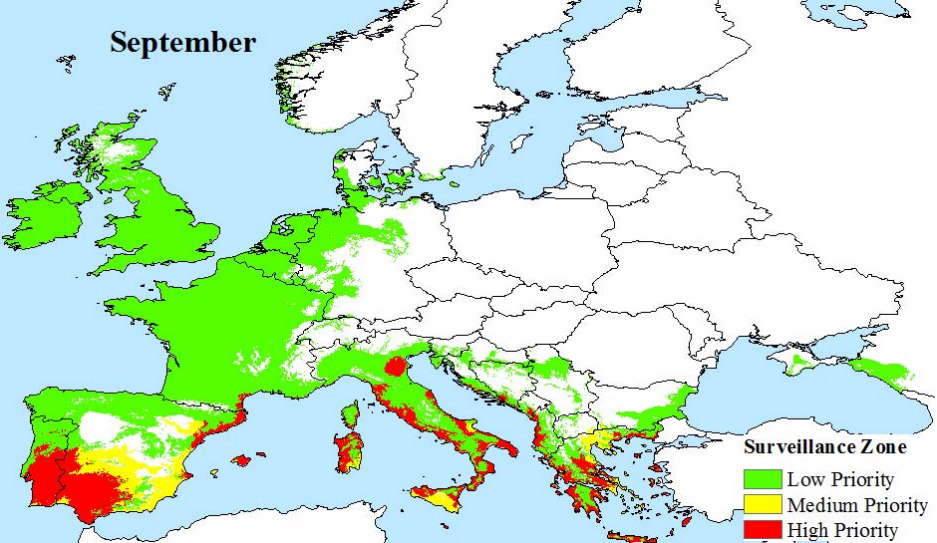
**Proposed European Chikungunya Surveillance Zones for September**.

The climatic limitations of *Aedes albopictus *and viral transmission across Europe, are mapped, illustrating that a large area of Europe is at risk from Chikungunya. Indeed, much of southern Europe has all the necessary conditions (High Priority Zone) to support Chikungunya transmission during the months of June (Figure 4), July (Figure 5), August (Figure 6) and September (Figure 7). Furthermore, this area of transmission risk could expand substantially (Medium Priority Zone) during an unusually warm summer or even farther afield (Low Priority Zone) should there be warmer than average years, as does occur periodically, and perhaps more frequently if many of the climate warming scenarios for Europe come to fruition.

The number of flights and estimated passenger numbers that each European country receives each year from Chikungunya active zones indicates that the majority of visitors are from India (Table 1). Germany and the UK appear to receive the most flights and therefore the highest volumes of passengers each year at 7.5 and 4.8 million passengers, respectively (Table 1), but their inbound visitors are less likely to be from Chikungunya active regions. Additionally, the surveillance maps illustrate that Germany and the UK are not high risk regions for Chikungunya transmission, as they are not generally warm enough to support developing adult *Aedes albopictus populations *(cf. Figures 4).

**Table 1:** Potential annual numbers of viraemic visitors to Europe have been extrapolated from a single week's FlightStats data [50]

**European Destination**	**Chikungunya Active Hotspots**	**Estimated Incidence (% passengers)**	**Estimated Annual**		**Potentially Viraemic Passengers**
			**Flights**	**Passengers**	
***France***	Reunion	35.71*	1,272	409,200	146,125
	Mauritius	0.81	1,488	497,040	4,026
	India	0.52	2,736	819,264	4,260
	Gabon	0.43	564	150,960	649
	Sri Lanka	0.20	156	36,864	74
	Congo	0.09	564	135,168	122
	Malaysia	0.01	1,020	307,200	31
			*12,240	**3,763,584**	**155,287**
					
***Germany***	Maldives	3.51	204	52,896	1,857
	Mauritius	0.81	204	54,528	442
	India	0.34	11,208	3,834,000	13,036
	Sri Lanka	0.20	216	54,048	108
	Malaysia	0.01	456	141,840	14
			**21,396**	**7,458,336**	**15,456**
					
***Italy***	Seychelles	10.81	144	40,608	4,390
	Maldives	3.51	264	61,440	2,157
	Mauritius	0.81	264	70,320	570
	India	0.01	492	151,200	15
	Malaysia	0.01	492	153,600	15
			**3,408**	**1,060,800**	**7,146**
					
***United Kingdom***	Mauritius	0.81	504	158,400	1,283
	India	0.35	6,360	211,136	739
	Sri Lanka	0.20	600	167,040	334
	Malaysia	0.01	1,080	398,112	40
			**18,864**	**4,824,368**	**2,396**
					
***Switzerland***	Maldives	3.51	48	12,288	431
	Mauritius	0.81	48	13,920	113
	India	0.24	2,304	610,512	1,465
			**6,144**	**1,858,304**	**2,009**
					
***Belgium***	India	0.19	3,025	878,976	1,670
	Congo	0.09	408	118,800	107
			**3,637**	**1,054,416**	**1,777**
					
***Netherlands***	India	0.20	2,136	610,512	1,221
	Malaysia	0.01	2,136	811,680	81
			**6,936**	**2,389,632**	**1,302**
					
***Spain***	Mauritius	0.81	480	13,920	113
			**936**	**184,560**	**113**
					

**Totals**			**73,561**	**22,594,000**	**185,486**

(*Note totals for each European country include visitors from other Chikungunya active regions, not listed as the estimated number of viraemic visitors was <1)

In terms of risk, the passenger volumes suggest that France and Italy are locations potentially of significant concern with 3.8 and 1 million inbound passengers each year from active regions, respectively. We have estimated the potential number of viraemic visitors this may represent, and this shows that France has the highest number, while Italy has the third highest number (Table 1). While these estimates are worrisome, they are also of limited usefulness due to the small sample used in creating this estimate, the potential complexity in unraveling flight patterns and visitor movements that are not direct from point to point. We urge that they be used with extreme caution, as is discussed below, and were intended as a preliminary analysis for the development of a more in-depth research project.

## Discussion & conclusion

This study provides an examination of important factors determining where and when potential outbreaks of Chikungunya are most likely to occur in Europe and proposes a time and geography varying system of surveillance zones. Results indicate:

i. A substantial area of Europe has suitable climate conditions for *Aedes albopictus *over-wintering that requires at least some, Low Priority, surveillance consideration.

ii. A large proportion of this proposed Low Priority Surveillance Zone has suitable temperatures for seasonal adult mosquito activity, but does not appear to have a climate that would allow for regular viral replication and transmission. We define this as a Medium Priority for surveillance because only the frequency of temperatures needed to support the virus are absent.

iii. Significant regions of Southern Europe do have temperatures necessary for viral transmission and they therefore define the High Priority surveillance zone.

iv. France and Italy have significant areas within the High Priority surveillance zone and may have the largest numbers of potentially viraemic visitors each year.

### Air travel: a bridge linking Chikungunya active zones to susceptible areas in Europe

A preliminary analysis of air travel from Chikungunya hotspots suggests that some 22.5 million passengers, of which 185,000 could be viraemic, are coming into Europe each year (Table 1). While the greatest number of air passengers may come from India, and the Italian outbreak of 2007 is believed to have originated via a viraemic visitor from India, the numbers of potentially viraemic passengers do not appear to be coming from India. The majority of French (imported) cases in are Comorian travellers returning to a well established community in Marseilles [62]. This is an example where a socio-culture factor, in this case post-colonial linkages, may be more important than the total passenger numbers in determining the risk of viraemic individuals igniting a local outbreak. EUROSTAT, the EU Statistical Bureau states that EU air traffic with India has increased by 57% between 2004 and 2006. The monthly International passenger traffic from India based on data by the Airport Authority of India indicates a growth of two million passengers per month in 2004 to three million passengers per month in 2007. However, note that visitors do not all pose the same risk, as is evidenced by India where the incidence of Chikungunya varies considerably and this is reflected in the regional risk within Europe. Eurosurvelliance [29] indicates that travellers from India could help ''fuel an epidemic'' in Europe, mainly due to the ''seasonal synchronicity'' of the vector and year round outbreaks of the disease. India, Kenya, Thailand and Reunion Island have all had outbreaks, which have coincided with the European summer.

### Limitations of the study

Information on global outbreaks, such as the recorded number of cases, are limited and this poses a problem when calculating the proportion of the population affected in each country, as they are unlikely to be temporally uniform. Our estimates of incidence (Table 1) did not take these factors into consideration. Also, surveillance data regularly under-report disease, for a variety of reasons including those cases that are asymptomatic; this is of particular concern because these cases can be viraemic, but feel well enough to travel. Consequently, notified case numbers may just be the 'tip of the iceberg'.

The spatial models developed here for probable vector distributions were based purely on temperature and precipitation, other factors were not considered. Although these climate variables are important, the absence of meteorological data specific to the individual outbreaks means that some refining of the proposed surveillance zones is possible. Additionally, the passenger traffic analysis is greatly limited by the absence of freely available data necessitating estimates being made using an aggregation of 3-day windows of flights available online from FlightStats [50]. Such an approach is susceptible to variations in travel patterns due to tourist fashion and global economic downturns. Additionally, while on average India may pose a great risk from imported viraemic cases, the pattern of imported French cases indicates the potential for future outbreaks to be driven, at least in part, by socio-cultural relationships, which are rarely factored into surveillance systems. Consequently, we cannot yet account for the seasonal nature of outbreaks. The numbers presented should only be used in relative comparisons.

### Further research

This study indicates that areas suitable for Chikungunya outbreaks would mainly be located in Southern Europe, during summer. Having identified where and when outbreaks are likely to occur, our results illustrate where further research is required in order to elicit information regarding other factors that would influence the quantification of risk within these broad regions. An up-to-date survey on the vector's current distribution is currently being developed by the ECDC [personal communication] and this should be compared with more complex models of vector survival in Europe. This information could enable health authorities to better geographically target their action to prevent further expansion of the vector's breeding sites across Europe.

A study of the virus-vector interaction within a European setting and also the competence of other European mosquitoes in transmitting the virus will prove beneficial to future modelling efforts. Models calculating the Reproduction number (R_0_) are now being proposed for the Chikungunya outbreaks that occurred on Reunion [63-65]. These models would allow us to quantify the risk across the currently defined "High Priority" surveillance zone described in this paper. A synthesis of such information could then enhance preparedness for possible outbreaks of Chikungunya.

Finally, the preliminary analysis of air passenger numbers with estimates of potential viraemic introductions has shown that much can be done, as the data does exist, although it is not generally accessible to most researchers. Analysis of this and other air industry databases could provide information on all airport-airport transfers and their intra- and inter-annual fluctuations. Furthermore, if these data were combined with national and regional tourism data, the flows of passengers to secondary destinations could be estimated (e.g. arrivals at Charles de Gaulle from Sri Lanka who then board domestic flights or trains to Marseilles) and a much better understanding of visitor movements from the regions of concern could be had.

## Competing interests

The authors declare that they have no competing interests.

## Authors' contributions

NT and CS conceived the study. NT performed the literature review and compiled data and maps under the supervision of CS. The first draft was produced by NT during her studies at Brunel University and all authors participated in the writing of subsequent drafts. All authors read and approved the final manuscript.

## References

[B1] Parida MM, Santhosh SR, Dash PK, Tripathi NK, Lakshmi V, Mamidi N, Shrivastva A, Gupta N, Saxena P, Babu JP, Rao PVL, Morita K (2007). Rapid and Real-Time Detection of Chikungunya Virus by Reverse Transcription Loop-Mediated Isothermal Amplification Assay. Journal of Clinical Microbiology.

[B2] Pialoux G, Gauzere BA, Jaureguiberry S, Strobel M (2007). Chikungunya, an epidemic arbovirosis. The Lancet infectious diseases.

[B3] Yadav JS (2006). A Special Issue on Chikungunya. ENVIS News Letter.

[B4] Carey DE (1971). Chikungunya and dengue: a case of mistaken identity?. Journal of the History of Medicine and Allied Sciences.

[B5] Diallo M, Thonnon J, Traore-Lamizana M, Fontenille D (1999). Vectors of Chikungunya virus in Senegal: current data and transmission cycles. The American journal of tropical medicine and hygiene.

[B6] Halstead SB, Scanlon JE, Umpaivit P, Udomsakdi S (1969). Dengue and chikungunya virus infection in man in Thailand, 1962-1964. IV. Epidemiologic studies in the Bangkok metropolitan area. The American journal of tropical medicine and hygiene.

[B7] Peyrefitte CN, Rousset D, Pastorino BA, Pouillot R, Bessaud M, Tock F, Mansaray H, Merle OL, Pascual AM, Paupy C, Vessiere A, Imbert P, Tchendjou P, Durand JP, Tolou HJ, Grandadam M (2007). Chikungunya virus, Cameroon, 2006. Emerging Infectious Diseases.

[B8] Peyrefitte CN, Bessaud M, Pastorino BA, Gravier P, Plumet S, Merle OL, Moltini I, Coppin E, Tock F, Daries W, Ollivier L, Pages F, Martin R, Boniface F, Tolou HJ, Grandadam M (2008). Circulation of Chikungunya virus in Gabon, 2006-2007. Journal of Medical Virology.

[B9] Myers RM, Carey DE (1967). Concurrent isolation from patient of two arboviruses, Chikungunya and dengue type 2. Science.

[B10] Seneviratne SL, Gurugama P, Perera J (2007). Chikungunya viral infections: an emerging problem. Journal of Travel Medicine.

[B11] Powers AM, Logue CH (2007). Changing patterns of chikungunya virus: re-emergence of a zoonotic arbovirus. Journal of General Virology.

[B12] Ross RW (1956). The Newala epidemic III. The virus: isolation, pathogenic properties and relationship to the epidemic. The Journal of Hygiene.

[B13] Kondekar S, Gogtay NJ (2006). Why Chikungunya is called Chikungunya. Journal of Postgraduate Medicine.

[B14] Rai S, Korane AK (2006). Chikungunya: The Emerging Epidemic, Review Articles. Bombay Hospital Journal.

[B15] Santhosh SR, Dash PK, Parida MM, Khan M, Tiwari M, Lakshmana Rao PV (2008). Comparative full genome analysis revealed E1: A226V shift in 2007 Indian Chikungunya virus isolates. Virus Research.

[B16] Schuffenecker I, Iteman I, Michault A, Murri S, Frangeul L, Vaney MC, Lavenir R, Pardigon N, Reynes JM, Pettinelli F, Biscornet L, Diancourt L, Michel S, Duquerroy S, Guigon G, Frenkiel MP, Brehin AC, Cubito N, Despres P, Kunst F, Rey FA, Zeller H, Brisse S (2006). Genome microevolution of chikungunya viruses causing the Indian Ocean outbreak. PLoS Med.

[B17] Saxena SK (2007). Re-emergence of the knotty Chikungunya virus: facts, fears or fiction. Future of Virology.

[B18] Queyriaux B, Simon F, Grandadam M, Michel R, Tolou H, Boutin J-P (2008). Clinical burden of chikungunya virus infection. The Lancet Infectious Diseases.

[B19] Pialoux G, Gaüzère BA, Jauréguiberry S, Strobel M (2007). Chikungunya, an epidemic arbovirosis. The Lancet Infectious Diseases.

[B20] (2007). Chikungunya Outbreak in Italy - Question and Answers. http://ecdc.europa.eu/en/press/news/Lists/News/ECDC_DispForm.aspx?List=32e43ee8-e230-4424-a783-85742124029a&ID=265&RootFolder=/en/press/news/Lists/News&MasterPage=1&PDF=true.

[B21] (2007). Chikungunya update: Outbreak in Italy, advice for travellers. Health Protection Agency.

[B22] Seyler T, Rizzo C, Finarelli AC, Po C, Alessio P, Sambri V, Ciofi ML, Atti D, Salmaso S (2008). Autochthonous Chikungunya virus transmission may have occurred in Bologna, Italy, during the summer 2007 outbreak. Eurosurveillance.

[B23] Hochedez P, Hausfater P, Jaureguiberry S, Gay F, Datry A, Danis M, Bricaire F, Bossi P (2007). Cases of chikungunya fever imported from the islands of the South West Indian Ocean to Paris, France. Eurosurveillance.

[B24] Medlock JM, Avenell D, Barrass I, Leach S (2006). Analysis of the potential for survival and seasonal activity of Aedes albopictus (Diptera: Culicidae) in the United Kingdom. Journal of Vector Ecology.

[B25] Straetemans M (2008). On behalf of the ECDC consultation group on vector-related risk for chikungunya virus transmission in Europe. Vector-related risk mapping of the introduction and establishment of Aedes albopictus in Europe. Eurosurveillance.

[B26] Fontenille D, Failloux AB, Romi R (2007). Should we expect Chikungunya and Dengue in Southern Europe?. Emerging pests and vector-borne diseases in Europe.

[B27] Chong J-R (2007). A tropical virus moves north.

[B28] (2007). Mission Report - Chikungunya in Italy, Joint ECDC/WHO visit for a European Risk Assessment. European Centre for Disease Prevention and Control.

[B29] (2008). Chikungunya virus in north-eastern Italy: a consequence of seasonal synchronicity. Euro Surveillance.

[B30] Lines J (2007). Chikungunya in Italy. Bristish Medical Journal.

[B31] Parola P, de Lamballerie X, Jourdan J, Rovery C, Vaillant V, Minodier P, Brouqui P, Flahault A, Raoult D, Charrel RN (2006). Novel Chikungunya virus variant in travelers returning from Indian Ocean islands. Emerging Infectious Diseases.

[B32] (2008). Cameroon, Climatological Information for Yaounde. World Weather Information.

[B33] (2008). Kenya, Climatological Information for Mombasa. World Weather Information Service.

[B34] (2008). Gabon, Climatological Information for Libreville. World Weather Information.

[B35] (2008). Thailand, Climatological Information for Khon Kaen. World Weather Information.

[B36] (2008). Singapore, Climatological Information for Singapore. World Weather Information.

[B37] (2008). Sri Lanka, Climatological Information for Colombo. World Weather Information.

[B38] (2008). India, Climatological Information for Ahmedabad, Bangalore, Chennai. World Weather Information Service.

[B39] (2008). Comoros, Climatological Information for Moroni. World Weather Information.

[B40] (2008). Mauritius, Climatological Information for Port Louis. http://www.worldweather.org/178/c01219.htm.

[B41] (2008). Port Blair, India: Climate, Global Warming, and Daylight Charts and Data. World Climate.

[B42] (2008). Climate, Reunion. World Travel Guide.

[B43] (2008). Climate Averages, Bologna. euro weather.

[B44] Hijmans RJ, Cameron S, Parra J, Jones P, Jarvis A, Richardson K (2006). WORLDCLIM Version 1.4, Current Conditions (1950 - 2000), ESRI grids. http://www.worldclim.org.

[B45] Knudsen AB (1995). Geographic Spread of Aedes albopictus in Europe and the concern among Public Health Authorities. European Journal of Epidemiology.

[B46] Eritja R, Escosa R, Lucientes J, Marquès E, Molina R, Ruiz S (2005). Worldwide invasion of vector mosquitoes: present European distribution and challenges for Spain. Biological Invasions.

[B47] Delatte H, Dehecq JS, Thiria J, Domerg C, Paupy C, Fontenille D (2008). Geographic distribution and developmental sites of Aedes albopictus (Diptera: Culicidae) during a Chikungunya epidemic event. Vector Borne Zoonotic Diseases.

[B48] Arca B, Lombardo F, Francischetti IM, Pham VM, Mestres-Simon M, Andersen JF, Ribeiro JM (2007). An insight into the sialome of the adult female mosquito Aedes albopictus. Insect Biochemistry and Molecular Biology.

[B49] (2008). Airport Information per country. TheAirDB.

[B50] (2008). Travel Tools: Flight Status, By Route. FlightStats.

[B51] (2007). Gabon: Chikungunya Virus in Libreville. DREF Bulletin No MDRGA001.

[B52] Depoortere E, Coulombier D, ECDC Chikungunya risk assessment group (2006). Chikungunya risk assessment for Europe: recommendations for action. Euro Survelliance.

[B53] (2007). Chikungunya- Fact Sheet, Latest update. European Centre for Disease Prevention and Control. http://ecdpc.europa.eu/Health_topics/Chikungunya_Fever/Disease_facts.html.

[B54] Krishna MR, Reddy MK, Reddy SR (2006). Chikungunya outbreaks in Andhra Pradesh, South India. Current Science.

[B55] Lahariya C, Pradhan SK (2006). Emergence of chikungunya virus in Indian subcontinent after 32 years: A review. Journal of Vector Borne Diseases.

[B56] de Lamballerie X, Leroy E, Charrel RN, Ttsetsarkin K, Higgs S, Gould EA (2008). Chikungunya virus adapts to tiger mosquito via evolutionary convergence: a sign of things to come?. Virology Journal.

[B57] Pastorino B, Muyembe-Tamfum JJ, Bessaud M, Tock F, Tolou H, Durand JP, Peyrefitte CN (2004). Epidemic resurgence of Chikungunya virus in democratic Republic of the Congo: Identification of a new central African strain. Journal of Medical Virology.

[B58] Raj GD, Rajanathan TMC, Parthiban M, Ramadass P (2007). Phylogenetic Characterisation of Chikungunya virus isolates from Chennai, Tamil Nadu, India. Current Science.

[B59] (2006). Chikungunya in India.

[B60] Prabhu N (2006). Over 78,000 cases of Chikungunya reported in state. The Hindu.

[B61] (2007). Controlling and Managing Chikungunya Fever Outbreak in Maldives, Window on SEAR.

[B62] (2005). Points de situation générale sur l'epidémie de chikungunya à La Réunion/Océan Indien.

[B63] Dumont Y, Chiroleu F, Domerg C (2008). On a temporal model for the Chikungunya disease: Modeling, theory and numerics. Mathematical Biosciences.

[B64] Boelle PY, Thomas G, Vergu E, Renault P, Valleron AJ, Flahault A (2008). Investigating transmission in a two-wave epidemic of Chikungunya fever, Reunion Island. Vector Borne and Zoonotic Diseases.

[B65] Bacaer N (2007). Approximation of the basic reproduction number R0 for vector-borne diseases with a periodic vector population. Bulletin of Mathematical Biology.

